# Terahertz Sensor via Ultralow-Loss Dispersion-Flattened Polymer Optical Fiber: Design and Analysis

**DOI:** 10.3390/ma14174921

**Published:** 2021-08-29

**Authors:** Wanli Luo, Peng Jiang, Qiang Xu, Lei Cao, Adam Jones, Kang Li, Nigel Copner, Yongkang Gong

**Affiliations:** 1 College of Physics and Optoelectronic Technology, Baoji University of Arts and Sciences, Baoji 721016, China; wanliluo73@126.com (W.L.); jiangpeng-548@163.com (P.J.); caolei1111@126.com (L.C.); 2 Engineering Technology Research Center for Ultrafast Optics and Advanced Material of Baoji, Baoji 721016, China; 3 Wireless and Optoelectronics Research and Innovation Centre, Faculty of Computing, Engineering and Science, University of South Wales, Cardiff CF37 1DL, UK; adam.jones@southwales.ac.uk (A.J.); nigel.copner@southwales.ac.uk (N.C.); 4 School of Physics and Astronomy, Cardiff University, Cardiff CF24 3AA, UK; Gongy10@cardiff.ac.uk

**Keywords:** polymer optical fiber, fiber optics sensors, terahertz wave, finite element method

## Abstract

A novel cyclic olefin copolymer (COC)-based polymer optical fiber (POF) with a rectangular porous core is designed for terahertz (THz) sensing by the finite element method. The numerical simulations showed an ultrahigh relative sensitivity of 89.73% of the x-polarization mode at a frequency of 1.2 THz and under optimum design conditions. In addition to this, they showed an ultralow confinement loss of 2.18 × 10^−12^ cm^−1^, a high birefringence of 1.91 × 10^−3^, a numerical aperture of 0.33, and an effective mode area of 1.65 × 10^5^ μm^2^ was obtained for optimum design conditions. Moreover, the range dispersion variation was within 0.7 ± 0.41 ps/THz/cm, with the frequency range of 1.0–1.4 THz. Compared with the traditional sensor, the late-model sensor will have application value in THz sensing and communication.

## 1. Introduction

The characteristics of the wide-spectrum, strong-penetration, high-security terahertz (THz) wave have all-important applications in THz tomography [1,2], detection technology [3,4], biomedical sensing [5,6,7,8,9], THz communication [10,11], polarization maintenance [12,13,14], nondestructive testing [15], and other fields. The THz functional device is based on the waveguide structure and is the premise to realize various application functions. It plays a crucial role in the development of a compact and powerful all-fiber THz system. 

In recent years, THz technology has made progress in optical fiber technology by realizing a variety of optical fiber functional devices, including the THz wave directional coupler [16,17], filter [18], beam splitter [19,20], optical switch [21], and the polarization controller [22,23]. Among them, the THz fiber device based on porous-core fiber has special advantages [24,25,26,27,28,29,30]. The appearance of PCFs was an overturning innovation in optical fiber technology because PCFs not only have all-time performance but also can overcome the inherent limitations of standard optical fibers [31]. Compared with conventional optical fibers, PCFs are flexible in design and can adjust transmission characteristics by controlling multiple geometric parameters. The light conduction mode of porous fiber is a total internal reflection mechanism. The microporous channel at the core of the PCF provides convenience for filling various functional materials for optical sensing [31,32,33,34].

However, a THz wave is easily absorbed by dielectric materials, so it is critical to select low-loss materials for THz wave transmission. The measures to reduce the material loss mainly include the selection of background materials and the novel structure of photonic crystal fibers (PCFs), such as porous- or hollow-core photonic crystal fibers [35,36,37]. There are many polymer substrate materials used in polymer fiber, including cyclic olefin copolymer (COC), polytetrafluoroethylene (Teflon^®^ or PTFE), polyethylene (PE), polyamide-6 (PA6), polycarbonate (PC), polymethyl methacrylate (PMMA), cyclo-olefin polymer (COP), high-density polyethene (HDPE), and so on [38,39,40,41,42]. COC is a new kind of optical polymer material whose commercial name is TOPAS^®^. It is remarkable that due to its amorphous structure, TOPAS^®^ has a tiny loss in the THz band, which is 1% of PMMA [43]. In recent years, the TOPAS^®^ microstructural fiber for THz transmission was reported frequently [44,45,46]. Therefore, TOPAS^®^-based POFs have opened up a new area of fiber sensing.

The rectangular porous-core POFs have powerful anisotropy which obtain outstanding optical transmission properties, such as flat dispersion, high birefringence, low loss, large numerical aperture, and the like. Therefore, based on the above discussion, we designed a TOPAS^®^-based terahertz sensor and used it to measure alcohol, combining the ethanol-filled rectangular microarray core and the modified hexagonal lattice cladding. As an important industrial raw material widely used in food, the chemical industry, the military industry, medicine and other fields [47,48], it is of great significance to determine the content of alcohol quickly, accurately, and sensitively.

For the complex structure of a POF-based THz sensor, the transmission characteristics of THz-PCFs are usually studied by the full-vector finite element method (FV-FEM) [49,50,51], the plane-wave method [52,53], an improved effective index method [54,55], the finite difference time domain method [56,57], the beam propagation method [58,59,60], and the multi-pole method [61,62,63]. Among these methods, the FV-FEM is best suited for calculating the transmission characteristics of optical fibers. FV-FEM technology has the advantages of a short computation time, less computational memory, and accurate calculation results.

In this work, a simulation analysis of a new-type TOPAS^®^-based THz sensor with a rectangular porous core is presented using the FV-FEM. The guiding properties, including birefringence, dispersion, confinement loss, and nonlinearity are studied thoroughly. It can be predicted that the ultrahigh relative sensitivity TOPAS^®^-based THz sensor has vast potential in the field of THz transmission and sensing.

## 2. Design Methodology

**Figure 1:**
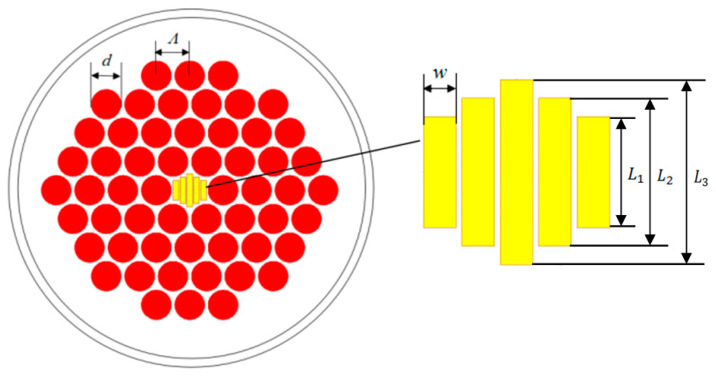
Cross-section of TOPAS^®^-based THz sensor. The figure shows an enlarged view of the rectangular porous core of the sensor. The diameter of the cladding air hole of the TOPAS^®^-based THz sensor is d, and the period is Λ. The width and length of the rectangular porous core are noted as w and L_i_ (i = 1, 2, 3).

Figure 1 shows a design model consisting of the ethanol-filled rectangular microarray core and modified hexagonal lattice cladding. The background material is TOPAS^®^. TOPAS^®^ is an amorphous, transparent copolymer with a cyclic olefin structure. Compared with other optical polymers, it has desirable properties, such as low density, high refractive index, high transparency, strong heat resistance, small coefficients of thermal expansion, stable chemical properties, strong acid and alkaline resistance, and high mechanical flexibility. The background material used in the design model has a refractive index of 1.53. The diameter of the cladding air hole of the TOPAS^®^-based THz sensor is *d*, and the period is Λ. We used ethanol with a refractive index of 1.354 as the analyte and filled it with a rectangular porous core. The width and length of the rectangular porous core are noted as *w* and L_i_ (i = 1, 2, 3). The extrusion, drilling–stretching, injection molding, and capillary stacking techniques, and the bulk polymerization process can be used to prepare POFs [64,65,66]. The advantage of these techniques is that the cross-sections of arbitrary shapes and size can be obtained in the preform [67]. These properties of TOPAS^®^ have opened up possibilities for sensing systems.

The FEM skillfully combines approximation theory, the partial differential equation, and variation and functional analysis. The principle of the FEM is to simplify complex problems into a series of simple problems, according to the variational principle, which are widely used in aerospace, mechanical engineering, optical engineering and other fields. Next, we introduced the basic theory of the FV-FEM in analyzing the transmission of electromagnetic waves in optical fibers.

Based on the anisotropic perfect matching layer (PML) condition, the following vector wave formula is obtained from Maxwell’s equations [49,50,51]

(1) $$\nabla \times \left({\left[s\right]}^{-1}\nabla \times E\right)-k_{0}^{2}n_{eff}^{2}\left[s\right]Ε=0$$

(2) $$\left[s\right]=\left[\begin{array}{ccc} s_{y}/s_{x} & 0 & 0\\ 0 & s_{x}/s_{y} & 0\\ 0 & 0 & s_{x}s_{y} \end{array}\right]$$

where *E* is the electric field, [*s*] is the perfect matching layer matrix, [*s*]^−1^ is a transposed matrix of [*s*], $n_{eff}$ is the refractive index, and *k*_0_ is the wavenumber in free space.

When the FV-FEM is used to simulate the TOPAS^®^-based THz sensor, a curvilinear hybrid nodal/edge unit is effective for precisely modeling curvilinear boundaries of holes and for avoiding spurious solutions. Dividing the cross-section of the TOPAS^®^-based THz sensor into some curvilinear hybrid nodal/edge units by the FEM, from Equation (1) we can obtain the standard eigenvalue equation

(3) $$\left[K\right]\left\{E\right\}=k_{0}^{2}n_{eff}^{2}\left[M\right]\left\{E\right\}$$

where {*E*} is the discretized electric field vector, and [*M*] and [*K*] represent the finite element matrices.

The relative sensitivity of a TOPAS^®^-based THz sensor represents the sensitivity response for the filling liquid, which can be expressed as [25]

(4) $$r=\frac{n_{r}}{n_{eff}}\times f$$

where *n_r_* and $n_{eff}$ are the refractive indexes of the analyte and the guided mode. The *f* is the percentage of the total power by holes power.

(5) $$f=\frac{\int_{sample}R_{e}\left(E_{x}H_{y}-E_{y}H_{x}\right)dxdy}{\int_{total}R_{e}\left(E_{x}H_{y}-E_{y}H_{x}\right)dxdy}\times 100$$

where *E_x_*_,*y*_ and *H_x_*_,*y*_ represent *x*- and *y*- components of the electric field and magnetic field, respectively.

The THz fiber with high birefringence can preserve the polarization of the lightwave in the fiber. It plays an extremely crucial role in the terahertz communication and sensing system, which requires a high-polarization state. The birefringence is expressed as [3]

(6) $$B=\left|n^{x}-n^{y}\right|$$

where *n^x^* and *n^y^* are the effective refractive indexes of the *x*- and *y*-polarizations, respectively.

Dispersion is an extremely vital index to characterize the transmission characteristics of the fiber. The dispersion *D*(*λ*) of the TOPAS^®^-based THz sensor can be obtained from the refractive index values vs. the wavelength by using [68]:

(7) $$D\left(\lambda \right)=-\frac{\lambda }{c}\frac{\partial^{2}\left|Re\left(n_{eff}\right)\right|}{\partial \lambda^{2}}$$

The effective mode area represents the area involved in the interaction between materials and light intensity. The effective mode area has applications in laser and communication devices and optical nonlinear effects [66].

(8) $$A_{\mathrm{eff}}=\frac{{\left(\iint {\left|E\right|}^{2}dA\right)}^{2}}{\iint {\left|E\right|}^{4}dA}$$

The confinement loss of the TOPAS^®^-based THz sensor is acquired from the following formula [16].

(9) $$Confinement\;loss=\left(\frac{4\pi f}{c}\right)Im(n_{eff})\;{[\mathrm{cm}}^{-1}]$$

The TOPAS^®^-based THz sensor with a large numerical aperture (*NA*) has application value in optical sensing [25].

(10) $$NA=\frac{1}{\sqrt{1+\frac{\pi A_{\mathrm{eff}}f^{2}}{c^{2}}}}$$

## 3. Simulation Results and Analyses

**Figure 2:**
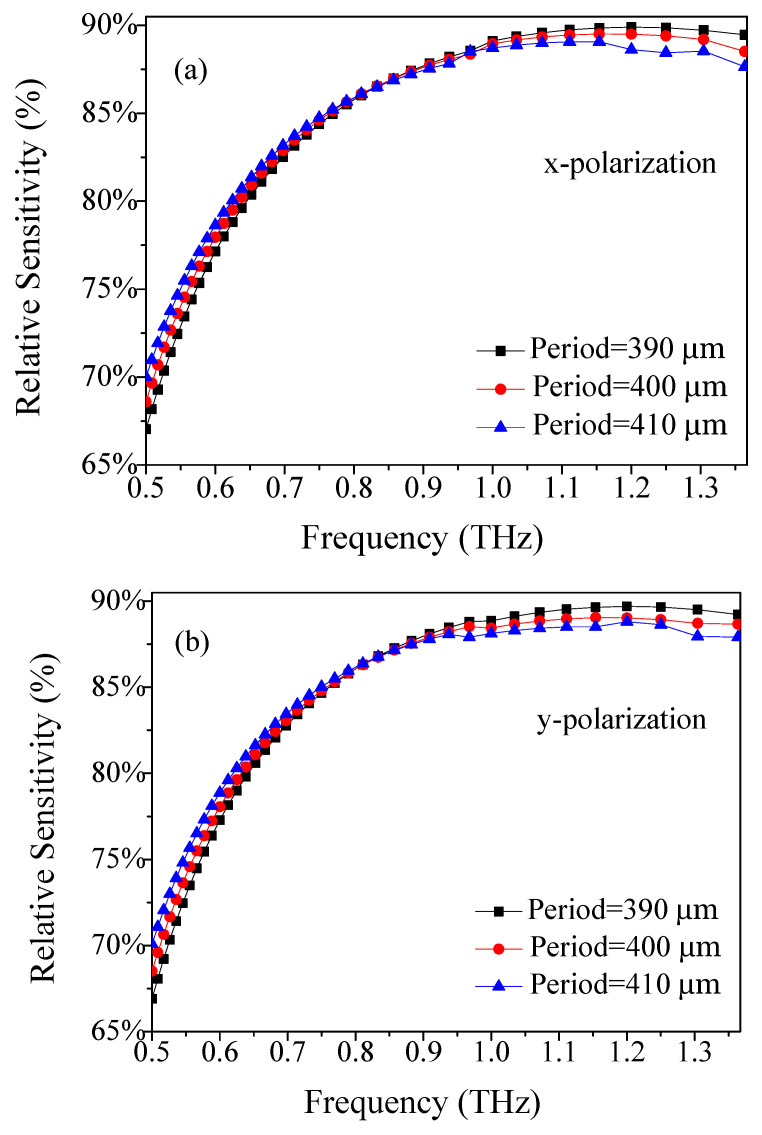
Frequency dependence of the relative sensitivity of (**a**) x- and (**b**) y-polarization for period Λ.

First, we analyzed the relation of the relative sensitivity of the TOPAS^®^-based THz sensor, with the frequency at period Λ = 390 μm, Λ = 400 μm, and Λ = 410 μm, where *d*/Λ = 0.90, *w* = 68 μm, as shown in Figure 2. It was noticed that the relative sensitivity was increased when the frequency was increased for a fixed Λ. The reason for this phenomenon is that light confinement reaches an optimum position at 1.2 THz, and that a further increase in frequency causes the useful light to leak towards the cladding and also to the material [25]. Figure 2a shows the relation of the relative sensitivity of the x-polarization with period Λ. It is evident that for (frequency is abbreviated as *f*) *f* < 0.81 THz, the value of relative sensitivity is higher for higher Λ, and for *f* > 1.03 THz, the value of the relative sensitivity is higher for lower Λ. Figure 2b shows a result similar to Figure 2a, as when f < 0.86 THz, the relative sensitivity of the y-polarization increases with the increase in period Λ, while when *f* > 0.91 THz, the relative sensitivity of the y-polarization reduces with the increase in period Λ. When f = 1.2 THz, the interaction between light and materials reaches its maximum *d*/Λ = 0.9, *w* = 68 μm and Λ = 390 μm. As the frequency increases further, the interaction between light and materials decreases and the sensitivity of the TOPAS^®^-based THz sensor decreases.

**Figure 3:**
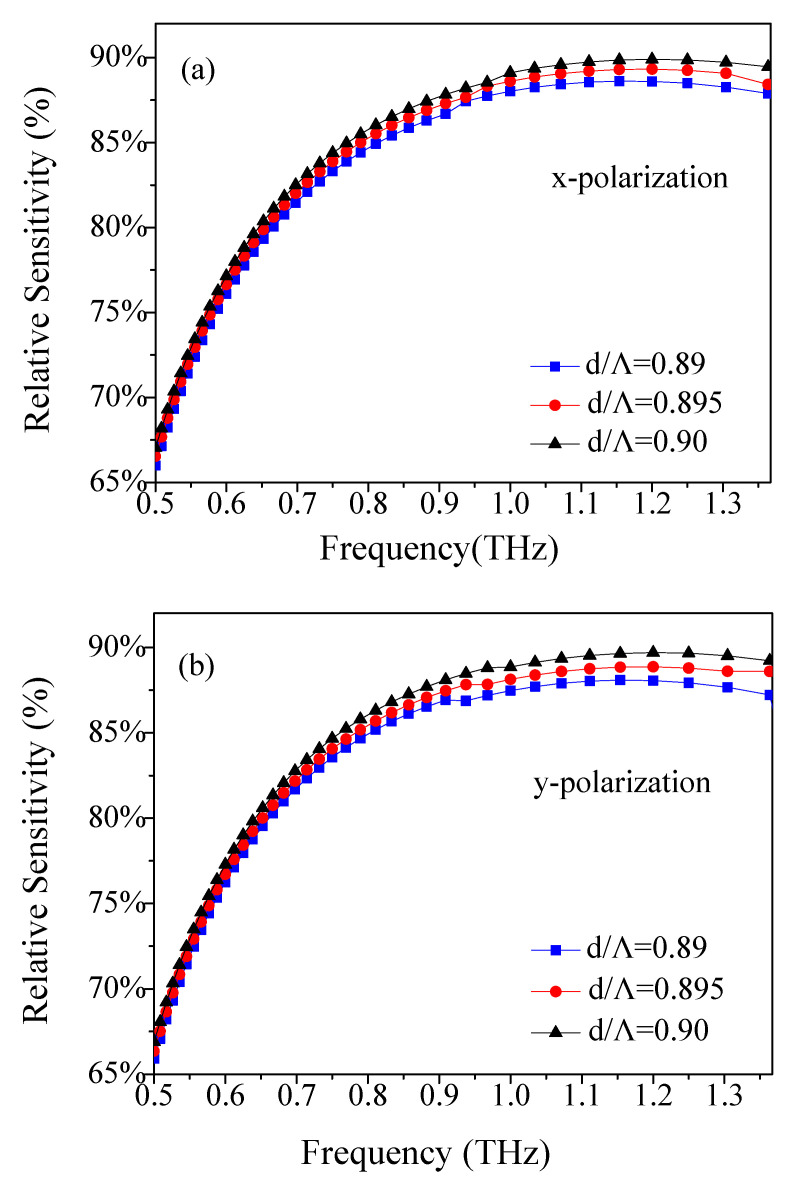
Frequency dependence of the relative sensitivity of (**a**) x- and (**b**) y-polarization for air–filling ratio *d*/Λ.

Next, by fixing Λ = 390 μm and *w* = 68 μm while changing the air–filling ratio *d*/Λ, the dependence of relative sensitivity of the TOPAS^®^-based THz sensor on frequency was further studied. The simulation results are shown in Figure 3. From Figure 3a,b, we noticed that the sensitivity increases at a certain air–filling ratio and then decreases. This is because, as the frequency increases, the effective refractive index of the guided mode increases but the core power fraction does not increase after a particular frequency. Moreover, the relative sensitivity of the TOPAS^®^-based THz sensor with the variation of *d*/Λ was investigated. It was observed that the sensitivity of the x- and y-polarizations are added to if *d*/Λ is increased because that also increases the core power fraction. When the frequency is 1.2 THz, the interaction between light and materials reaches its maximum, at Λ = 390 μm, *w* = 68 μm and *d*/Λ = 0.9. As the frequency increases further, the interaction between light and materials decreases and the sensitivity of the TOPAS^®^-based THz sensor decreases.

**Figure 4:**
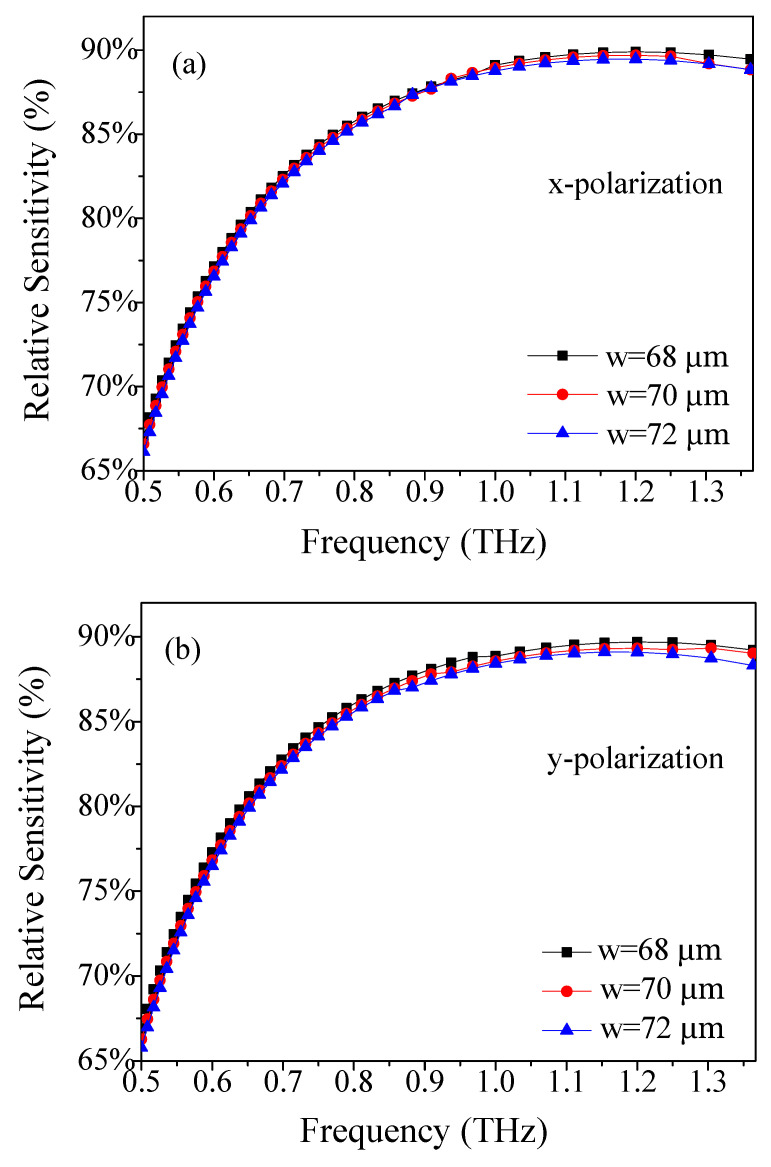
Frequency dependence of the relative sensitivity of (**a**) x- and (**b**) y-polarization for the width of rectangular porous core (w).

Additionally, we fixed Λ = 390 μm, and *d*/Λ = 0.90 and found that when the width of the rectangular porous-core (w) is varied, the dependence of the relative sensitivity of the x- and y-polarizations on frequency are also varied, as depicted in Figure 4. From Figure 4a,b, it is found that the trend dependence on the frequency of the three curves is similar and close to each other. When the frequency is 1.2 THz, the interaction between light and materials reaches its maximum at Λ = 390 μm, *d*/Λ = 0.9, and *w* = 68 μm. This phenomenon can be interpreted as the following: the core power fraction may be closely related to the amount of analyte filled inside the core holes.

**Figure 5:**
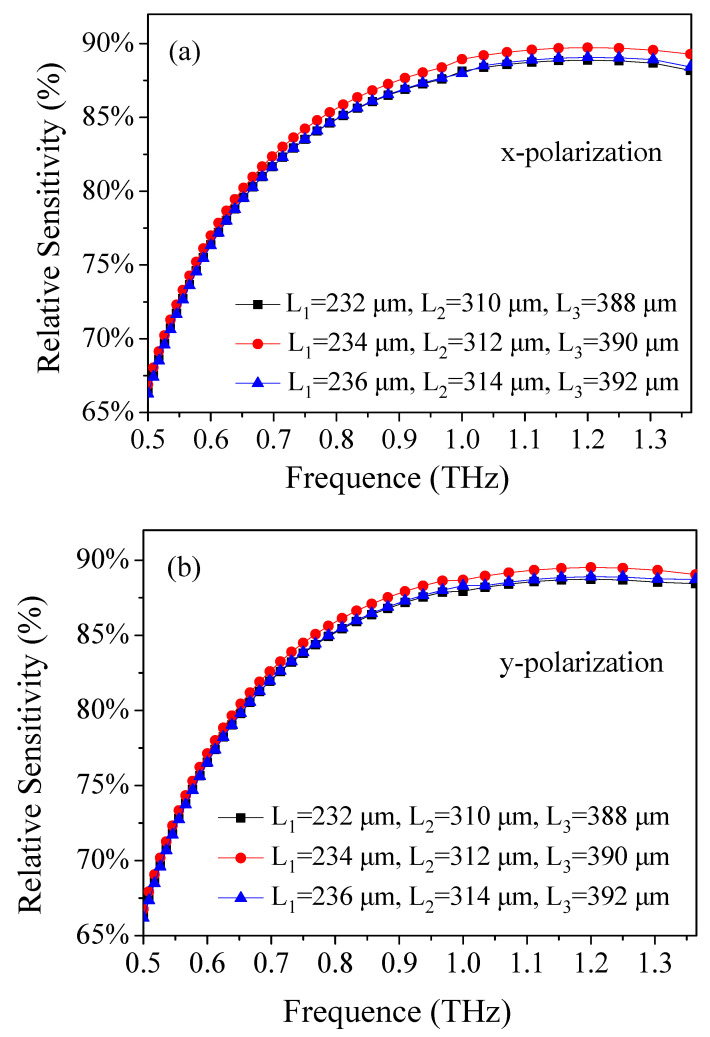
Frequency dependence of the relative sensitivity of (**a**) x- and (**b**) y-polarization for the length of rectangular porous core (L_i_).

The performance of relative sensitivity with the variation of the length of rectangular porous core (L_i_) was also investigated, and is shown in Figure 5. It is found that the relative sensitivity of the x- and y-polarizations increase first and then decrease with L_i_. When the frequency is 1.2 THz, the interaction between light and materials reaches its maximum, at Λ = 390 μm, *d*/Λ = 0.9, *w* = 68 μm, L_1_ = 234 μm, L_2_ = 312 μm, and L_3_ = 390 μm. The reason may be closely related to the amount of analyte filled inside the core holes, which affects the core power fraction. Under the optimum design conditions, the relative sensitivity of the TOPAS^®^-based sensor in the x- and y-polarization modes at the frequency of 1.2 THz is 89.73% and 89.52%, respectively. The relative sensitivity of the proposed TOPAS^®^-based THz sensor is much higher than sensors in the references [25,69,70,71,72,73,74,75,76]. The primary causes for the high sensitivity of the proposed TOPAS^®^-based terahertz sensor are related to the selection of background materials (TOPAS^®^), the design of new fiber structures (modified hexagonal lattice cladding and ethanol-filled rectangular microarray core), and the filling of functional materials (ethanol).

**Figure 6:**
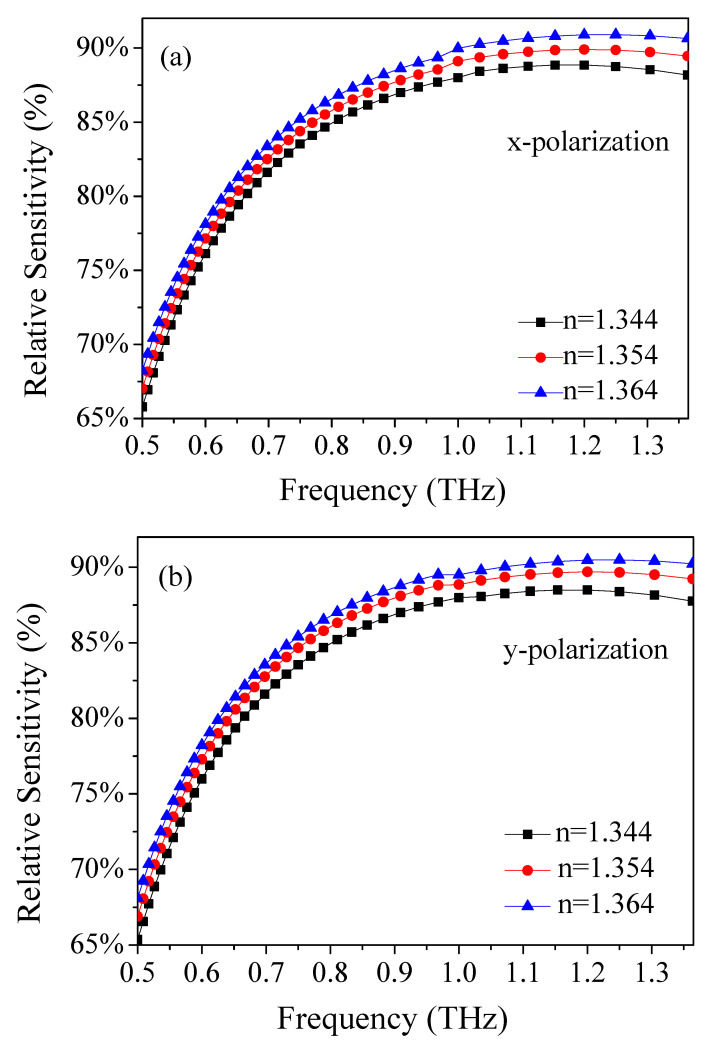
Frequency dependence of the relative sensitivity of (**a**) x- and (**b**) y-polarization for refractive index of analyte.

In order to detect the amount of ethanol in food and various environments simply and efficiently, we changed the effective refractive index of analyte in the porous core. Figure 6a,b illustrate the relative sensitivity of the x- and y-polarizations with the frequency at a different effective refractive index. It was found that the maximum relative sensitivity is obtained at *n* = 1.364. Meanwhile, we also observed that the tendency dependence of the three curves on the frequency is similar, and the difference is very obvious. The above results indicate that the sensor is very sensitive to the analyte measurement.

**Figure 7:**
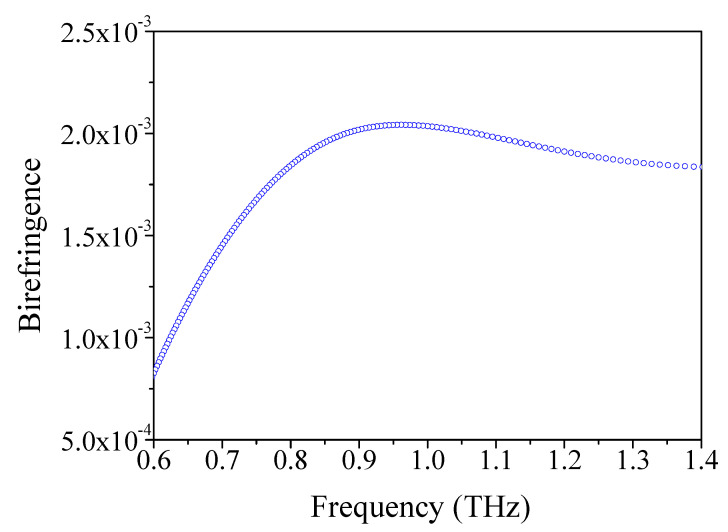
The relation of birefringence with frequency.

High-birefringence fibers have application value in optical communication, fiber sensors, and high-precision optical instruments. Figure 7 reveals the variation of the birefringence as a function of frequency under optimum conditions. It was found that the birefringence is sensitive to the varying frequency. It was seen that the birefringence is about 1.91 × 10^−3^ at the frequency of 1.2 THz, which is comparable to previous reports [16,68,73,77]. The high birefringence of the sensor can be obtained by introducing asymmetric defects such as a porous core. This kind of high-birefringence TOPAS^®^-based THz fiber with a porous core provides a new scheme for THz polarization controllers.

**Figure 8:**
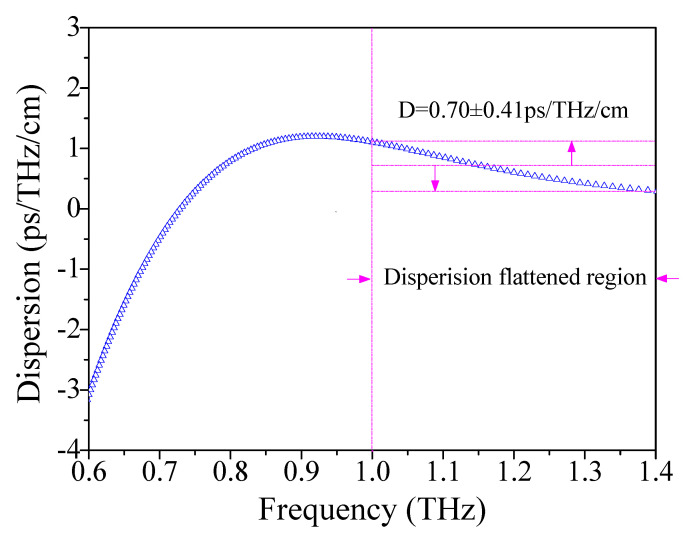
The relation of dispersion with frequency.

The technical problems to be considered in designing THz waveguides are mainly to realize low loss and flat dispersion in THz transmission, so as to replace the control of THz transmission of traditional optical devices in free space, and to finally promote the development of a compact THz system. Additionally, Figure 8 shows the properties of dispersion in the frequency domain for the optimum conditions Λ = 390 μm, *d*/Λ = 0.90, and *w* = 68 μm. It was observed that the dispersion variation is within 0.7 ± 0.41 ps/THz/cm in the frequency domain of the 1–1.4 THz range, which is lower than the references [10,78].

**Figure 9:**
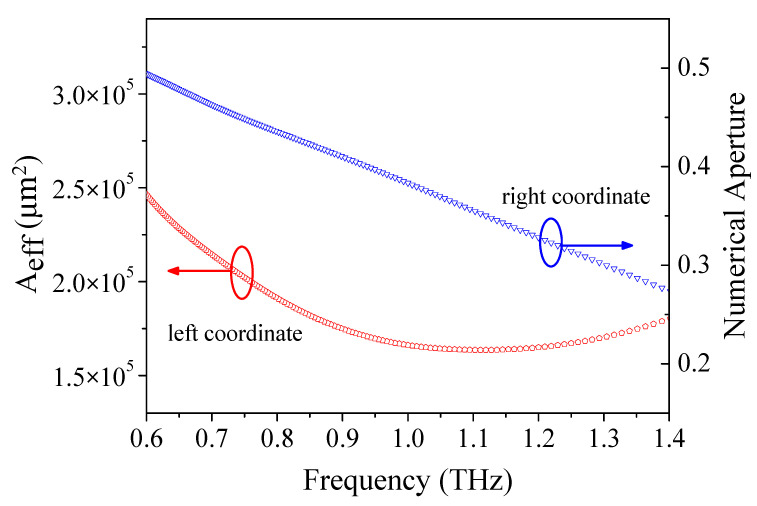
The relation of effective mode area and numerical aperture with frequency.

A low effective mode area is applicable to optical nonlinear effects, while a large effective mode area is applicable to laser communication and optoelectronic devices. The numerical aperture (*NA*) is a crucial physical quantity of the TOPAS^®^-based terahertz sensor, which can be obtained by adding the refractive index difference between the core and the cladding of the POF. As shown in Figure 9, we simulated the effective mode area and *NA* in the frequency domain for the optimum design conditions Λ = 390 μm, *d/*Λ= 0.90, and *w* = 68 μm. It was distinctly discovered that the effective mode area decreases in the low-frequency domain and then increases in the high-frequency domain. Moreover, from the results, the effective mode area is 1.65 × 10^5^ μm^2^, and the numerical aperture is about 0.33 at 1.2 THz. However, most previously designed sensors ignored the effective mode area [69,70,76,77,78,79,80,81] and numerical aperture [10,71,72,73,74,75,76,78,79,80,81] of the PCF.

**Figure 10:**
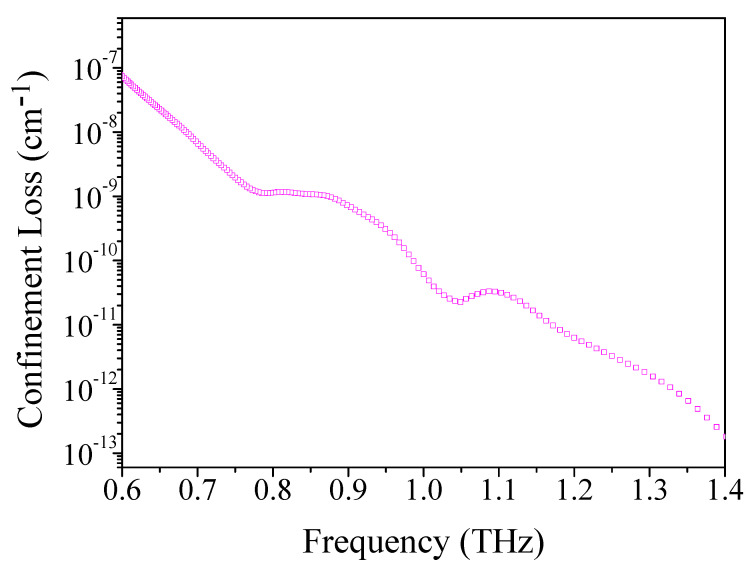
The relation of constraint loss with frequency.

The ultralow-loss THz fibers with the flattened dispersion have an important application prospect in imaging, sensing, communication, and nondestructive testing. Based on the function device of the low-loss THz waveguide, the necessary components of the THz system were constructed. Figure 10 illustrates the confinement loss of the suggested TOPAS^®^-based THz sensor for the optimum design parameters. It can be found that the confinement loss decreases with the increase in frequency. The reason for the phenomenon is that the guided mode is powerfully constrained in the position of the rectangular porous core in a high-frequency domain. It can be seen that the THz fiber with a rectangular porous core integrates subwavelength air holes with a high duty ratio, which can reduce the material absorption loss well. The simulated confinement loss is 2.18 × 10^−12^ cm^−1^, which is better than the previous references at optimal conditions [10,25,70,71,72,74,75,78,79].

**Figure 11:**
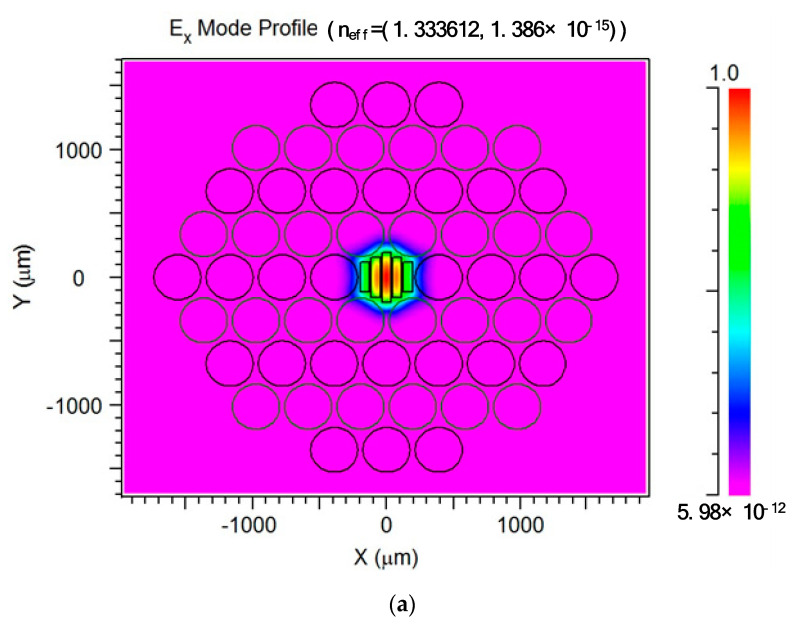

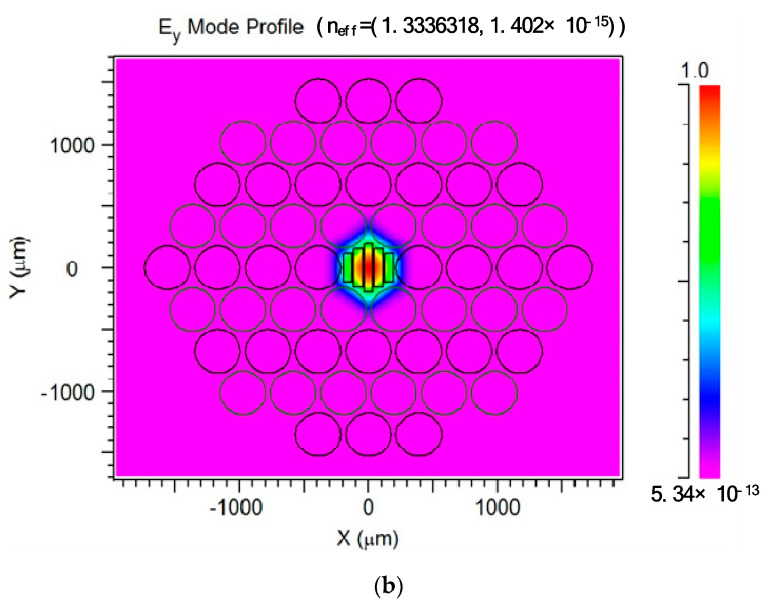
Mode field distribution of x- (**a**) and y-polarization (**b**) for the suggested TOPAS^®^-based THz sensor. The circles are a cross-section of the air holes in the cladding.

Figure 11 shows the mode field distribution of the *x*- and *y*-polarizations for the suggested TOPAS^®^-based THz sensor, when Λ = 390 μm, *d*/Λ = 0.90, and *w* = 68 μm. As can be seen from Figure 9, the light field is firmly restrained at the core of POF, indicating that the rectangular porous core produces an index of discrepancy between the *x*- and *y*-polarization modes based on the anisotropy of POF.

## 4. Conclusions

In summary, we numerically investigated a new TOPAS^®^-based THz sensor in the THz region. At optimal design parameters, the sensor exhibits an ultrahigh relative sensitivity of 89.73%, an ultralow confinement loss of 2.18 × 10^−12^ cm^−1^, a high birefringence of 1.91 × 10^−3^, a large numerical aperture of 0.33, and the flattened dispersion of 0.7 ± 0.41 ps/THz/cm. Moreover, it is feasible to fabricate the sensor by using the existing optical fiber fabrication technology. Therefore, based on the excellent sensing characteristics and design flexibility of the TOPAS^®^-based THz sensor, these results will create a new window for next-generation THz technology and will play a significant role in the food industry, environmental science, safety monitoring, biomedical industry, and other fields. 

## Data Availability

The data presented in this study are available on request from the corresponding author.
